# Expressed Breast Milk Analysis: Role of Individualized Protein Fortification to Avoid Protein Deficit After Preterm Birth and Improve Infant Outcomes

**DOI:** 10.3389/fped.2021.652038

**Published:** 2022-01-13

**Authors:** Sharmeel Khaira, Antoinette Pert, Emily Farrell, Cecelia Sibley, Karen Harvey-Wilkes, Heber C. Nielsen, MaryAnn V. Volpe

**Affiliations:** ^1^Newborn Medicine, Tufts Children's Hospital, Tufts Medical Center, Boston, MA, United States; ^2^Tufts University School of Medicine, Boston, MA, United States

**Keywords:** breast milk, preterm infants, milk analysis, growth, neurodevelopment

## Abstract

**Background:** Expressed breast milk (EBM) protein content is highly variable between mothers and often below published values that are still used for EBM protein fortification strategies. This approach may result in significant protein deficit and suboptimal protein energy (P/E) ratio. The study aim was to determine whether individualized EBM protein analysis and fortification will reduce preterm infant protein deficits and improve growth and neurodevelopmental outcome.

**Study Methods:** In a single-center randomized, blinded study of infants born at 24 0/7–29 6/7 weeks, mother-specific protein values measured by a milk analyzer were used to individualize infant-specific protein intake (interventional group, IG), and compared this to a standardized protein fortification scheme based on published values of EBM protein content of 1.4 g/dL (control group, CG). For IG, milk analyzer protein values of mother's EBM were used to adjust protein content of the EBM. The CG EBM protein content was adjusted using the standard published value of 1.4 g/dL and not based on milk analyzer values. EBM protein content, protein intake, protein/energy (P/E) ratio, weight (WT), head circumference (HC), length (L), growth velocity (GV) from 2 to 6 weeks of age, WT, HC and L Z-Scores at 32- and 35-weeks PMA, and lean body mass (35 weeks PMA skin fold thickness) were measured. Neurodevelopment was assessed by Bayley III at average 24 months corrected gestational age (CGA).

**Results:** EBM protein content before fortification was significantly below published values of 1.4 g/dL at all time points in both CG and IG. CG protein deficit was significantly decreased and progressively worsened throughout the study. Individualized protein fortification in IG avoided protein deficit and optimized P/E ratio. Although no significant change in short-term GV (at 6 weeks of age) was seen between groups, IG infants born at <27 weeks had significant improvements in WT and L z-scores, and leaner body mass at 32 and 35 weeks PMA. IG exhibited significantly improved cognitive scores at 24 months CGA.

**Conclusions:** Infant-specific protein supplementation of mother's EBM optimized P/E ratio by eliminating protein deficit and improved growth z scores at 32- and 35-weeks PMA and neurocognitive testing at 24 months.

## Introduction

It is well known that expressed breast milk (EBM) is superior to formula feeding for preterm infants due to its effects on organ maturation, the immune system, and gastrointestinal function (1–5). Preterm infants fed human milk have better tolerance of enteral feeds and reach full enteral feeds faster than formula fed infants (6). While breast milk has these positive effects, at the volume restriction levels needed for preterm infants, its protein content does not meet these infants' high nutritional requirements (7–11). Thus, protein intake from EBM can be substantially below preterm infant protein requirements. This requires supplementing EBM with protein using commercially available human milk fortifiers in order to achieve adequate protein/energy ratios (3, 7, 11).

Traditionally, neonatal intensive care units (NICU) feeding protocols and commercially produced human milk fortifiers have used published mean values of EBM protein levels to estimate the additional protein amount with which to fortify EBM. These sources treat all breast milk as having uniform protein content (1.4 g/dL protein) and use this value when adjusting the protein content of EBM to achieve a specified protein/energy ratio. It is now known that this value is only relevant to early postnatal milk protein content (10, 12, 13). Not only does EBM protein content decrease significantly over time of lactation, but breast milk from individual mothers is very diverse across a given NICU population (8, 11, 14, 15). Using a single mean protein value has ignored this mother to mother and lactation variability and can result in suboptimal protein intake (3, 11, 16, 17).

Growth and, more specifically, attainment of appropriate lean body mass, is one of the most important factors impacting favorable outcomes in extremely low birth weight (ELBW) infants (18, 19). Unfortunately, by 36 weeks corrected gestational age (CGA), many ELBW infants exhibit postnatal growth restriction (<10 % for weight and length) despite being appropriate for gestational age (AGA) at birth (2, 7, 19–24). Especially for infants born at <30 weeks' gestation, protein deficits after preterm birth from inadequate protein intake along with increased protein losses at a time in development notable for high levels of protein accretion leads to this growth failure and inadequate lean body mass (9, 25–27).

Studies have shown that 3.5–4.5 g/kg/day protein intake and optimized protein energy (P/E) ratio of 3.2–4.1 g/100 kcal improves lean body mass accretion and limits fat deposition while assuring adequate symmetric growth (7, 13). This has led to the use of either standardized fortification strategies by adding a fixed amount of fortifier with a known amount of protein to mother's EBM, or adjustable fortification strategies with the amount of added protein fortifier being based on BUN levels as an indirect measure of protein sufficiency or insufficiency (2, 3, 16, 28). Using a mid-infrared milk analyzer, we previously showed that there was significant variability in protein content between EBM from different mothers. This milk analysis identified that the “actual” infant protein intake was 10% lower than the “estimated or presumed” intake based on the measured amounts of protein being added to the EBM using published EBM protein levels in an attempt to bring total daily protein intake to 4.5 g/kg/day (8). Such discrepancies between the “actual” and “estimated” protein intake helps to explain why more aggressive preterm infant nutritional practices have not eliminated extrauterine growth failure. This problem continues to place preterm infants at risk for poor neurodevelopmental outcomes and strongly suggests that common preterm infant nutritional care practices for protein supplementation remain inadequate (7, 29, 30).

To address this area of nutritional concern for preterm infants, we designed a randomized, blinded study to examine the potential value of individualized protein supplementation based on direct measurements of each mother's EBM protein content. We hypothesized that using mother-infant specific analysis of mother's EBM protein content followed by infant-specific supplementation of protein would reduce the “protein deficit” experienced by infants during their recovery period after preterm birth, thus optimizing P/E ratio and subsequently improving the quality of their postnatal growth and neurodevelopmental outcome.

## Methods

### Participants

IRB approval was obtained from the Tufts Health Sciences Institutional Review Board prior to initiation of the study. All infants meeting the inclusion criteria of gestational age <30 weeks gestation at birth, admission to the Tufts Medical Center Level III NICU, and the mother's intention to provide exclusive EBM enteral feedings for her infant were eligible. Infants with major congenital anomalies and/or known chromosomal abnormalities that could affect growth were excluded. Eligible participants born between 2012 and 2014 were prospectively identified through the Tufts Medical Center NICU admission log. Informed consent was obtained from each subject's parents.

### Study Design

**Figure 1:**
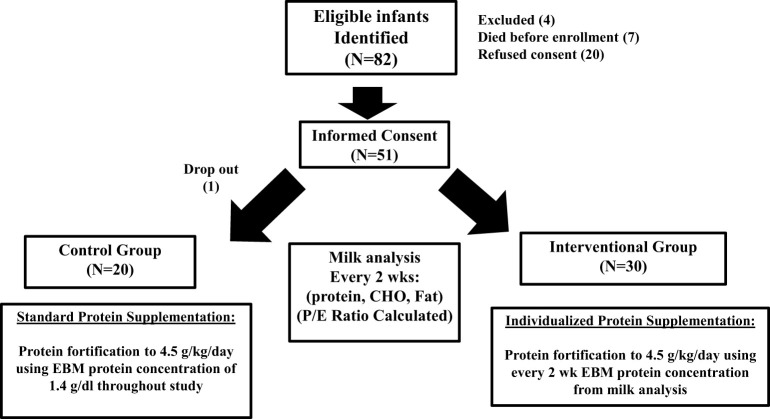
Flow chart showing the study population.

Figure 1 outlines the study population and study schematic. Randomization scheme was done by a predetermined block design to equally stratify subjects from 24 to 29 weeks gestation at birth between the Control Group (CG) and the Interventional Group (IG).

Infants in the CG received standard protein fortification using published values for EBM protein content. Infants in IG received individualized protein fortification using results of their mother's EBM protein content from the milk analysis to fortify the EBM as described below. Multiple births (twins, triplets) were randomized together based on our desire to use the identical feeding practice for siblings. This was chosen to minimize the risk of accidental protocol violation, and the perception that parents prefer to have their children receive equal treatment.

The primary outcome measure of this prospective study was to determine whether individualized protein supplementation based on the specific mother's EBM protein content, compared to standard EBM fortification as used in our NICU, led to a difference in growth, as measured by the velocities of weight gain (g/kg/day), head circumference gain (cm/week), and length gain (cm/week). The secondary outcome was neurodevelopmental status at 24 months postnatal age. We chose growth as the primary outcome as it is an important factor impacting improved outcomes in ELBW infants.

We powered our study based on previously published data from our NICU in which the average weight growth velocity of infants born at <30 weeks' gestation was 13.8 g/kg/day between DOL 14–42 (31). Based on this information and the knowledge that weight growth velocity of at least 16–17 g/kg/day along with increased protein intake and optimized protein/energy ratio in the early weeks after preterm birth is associated with improved neurodevelopmental outcome, we powered our study to test the hypothesis that improved protein intake using the individualized milk analysis would increase growth velocity by 3.5 g/kg/day (18, 22, 25, 26, 30–36).

We prospectively calculated growth velocity at DOL 42 (6 weeks of age) based on our prior study (31). In addition, this stopping point was determined by the fact that the oldest infants in the cohort (those born at 29–30 weeks' gestation) would reach 35–36 weeks corrected gestational age at this time point and likely be ready for discharge. Determining this stopping rule in advance allowed us to have complete growth velocity data on the whole cohort.

All investigators were blinded to subject allocation except the NICU nutritionist. The NICU nutritionist was responsible for coordinating EBM protein measurements and for calculating the amount of supplement to add to the EBM for all infants in the study. We prospectively planned the following data analysis process. First, the results for the whole cohort (24–29 6/7 weeks gestational age at birth) were analyzed. This was followed by an analysis of the youngest half of the cohorts (<27 weeks gestational age) to gain further insight into how nutritional needs and postnatal growth are impacted by gestational age at birth. These infants are at higher risk for postnatal growth failure than infants born at later gestational ages.

### Milk Analysis

EBM protein content was measured using the Julie Z7 Automatic Milk Analyzer (Scope Electric Ltd, Germany) at day of life (DOL) 10 and every 2 weeks thereafter until discharge to home or transition to Level two nurseries (33–35 weeks PMA) (8). The number of milk analyses per infant, therefore, ranged from 2 to 7 analyses per infant depending on the gestational age at birth. The decision to perform milk analysis every two weeks was based on the experience and information from a previous study by us and others (8) to capture post-natal variation in breast milk protein content in individual mothers while limiting the times that breast milk would have to be used for analyses and thus removed from the total supply provided by each mother to their infant. The Julie Z7 mid-infrared analyzer was the device used at donor milk banks across the country at the time of this study. This device is calibrated by Scope Electric (company producing the device) for human milk macronutrient levels and was validated by the company to have a range and accuracy as follows: (1) Protein = 0–15.00 ± 0.01 mg/100 mL, (2) Fat = 0–50.00 ± 0.01 mg/100 mL, and (3) Lactose = 0–21 ± 0.01 mg/100 mL. Further, prior to the start of the study we validated the accuracy of the measurement, using EBM samples with protein additive mixed using our mixing protocol that confirmed consistent and reproducible results from 10 ml milk samples used for the analysis. Five samples tested from the same EBM batch had EBM protein range of ± 0.01 g/dL.

EBM samples were not taken for analysis in the first 10 days of life, to not deprive infants of colostrum or any of their mother's milk supply between DOL 1 and DOL 10. Thereafter, EBM protein content was measured at 2-week intervals. At each analysis time point, the EBM collected over the preceding 24-h period was placed into sterile containers after pumping by each mother and brought to the NICU unfrozen without homogenization. Since the expressed breast milk was from the infant's own mother, it was not pasteurized. Using a designated sterilized work surface (Sani-Cloth Plus, PDI Inc., Orangeburg, NY), the 24-h EBM collection was pooled at room temperature and thoroughly mixed by gently pouring the milk between several large sterile mixing bottles, followed by removal of a 10-mL sample that was placed in a subject-coded and labeled test-tube as previously reported (8). By using a pooled 24-h, mixed EBM collection from which to withdraw the 10 mL aliquot for analysis, the circadian and pump time variability in breast milk macronutrient content was minimized, given a more comprehensive knowledge of the breast milk content in that total 24-h period. The remainder of the pooled sample was returned to the original containers and labeled with the receipt date to match with milk analysis. The 10-mL aliquot represented <1% of a 24-h collection of milk, thus a very minimal amount of an individual infant's expressed milk was lost to analysis. The 10-ml aliquot was then analyzed at room temperature within 24 h using the Julie Z7 Automatic Milk Analyzer and protein, fat, and lactose concentrations were recorded as g/dL. Total nutrient energy (kcal/dL) was calculated using standard values of 4 cal/g for protein, 9 cal/g for fat, and 4 cal/g for lactose, and the results used to calculate protein/energy (P/E) ratios. The calculated P/E ratios for each 2-week period were based on the most recently available milk analysis (protein, fat, and carbohydrate content) and the known amount of human milk fortifiers being added to the breast milk in the control and interventional groups.

### Fortification Scheme

The details of feeding advancement in both groups were as follows. Based on nutritional guidelines in our NICU at the time of the study, all subjects were advanced on NICU feeding protocols with EBM feedings initially at low volumes that were increased daily in a stepwise fashion as tolerated. When the study participants (both CG and IG) reached 100 mL/kg/day of enteral feeding, the EBM was fortified with liquid human milk fortifier (HMF, Abbott Laboratories, Columbus, OH) to increase EBM calories stepwise, first to 22 and then to 24 cal/oz over 48 h and to final enteral feeding volume of 150 mL/kg/day and 120 cal/kg/day. This provided the same fractional supplementation of protein and total calories to infants in both groups up until this point.

Additional protein fortification above the amount in the HMF was done using liquid protein fortifier (LP, Abbott Laboratories, Columbus, OH) to bring the protein intake to the target level of 4.5 g/kg/day. At the point of adding additional protein from LP was when the nutritional intake differed between the CG and IG. Therefore, the only difference in adjustment of energy intake between the CG and IG groups was the amount of supplemented protein provided as LP.

The protein supplementation in CG vs. IG was as follows. Subjects in the CG received standard protein fortification to bring EBM protein content (with LP) up to an assumed 4.5 g/kg/day based on the published EBM protein content of 1.4 g/dL, which was the most recent data at the time of study (12). For subjects in IG the biweekly EBM protein analysis results were used to calculate the amount of supplemental protein (using LP) needed to achieve an intake of 4.5 g/kg/day. No supplementation for the CG infants was based on an analysis of the mother's EBM. All calculations and supplementation levels were determined by the NICU nutritionist (who was a co-investigator on the study), who instructed the nursing team on the amount of protein supplement to provide for each infant.

If the weight gain of any infant was deemed inadequate by the healthcare team while receiving 24 cal/oz EBM fortified with HMF and supplemental protein with LP, further calories were provided by additional supplementation of the EBM with MCT oil to further increase the caloric content in 2 cal/oz increments. Any changes in caloric intact were accounted for in the study data by the protein/energy (P/E) ratio evaluations. The fortification scheme was maintained in all subjects until 34–35 weeks gestation at which time infants were transitioned off HMF and additional protein to a compatible feeding regimen for discharge from hospital to home. Therefore, the duration of time in study depended on gestational age at birth and ranged from 4 to 5 weeks (for the infants born at 29 weeks gestation) to 10 to 11 weeks (for infants born at 24 weeks gestation).

### Clinical Information

The following information was obtained from maternal records: Maternal steroid administration and number of doses, presence of chorioamnionitis (37, 38), preeclampsia, and diabetes. Information concerning postnatal course and management was obtained from each infant's medical records, including: serum creatinine, blood urea nitrogen (BUN) and CO_2_, postmenstrual age at discharge and diagnoses of necrotizing enterocolitis (Modified Bell's Staging Criteria, Stage 2 or greater), culture proven sepsis, bronchopulmonary dysplasia (39), retinopathy of prematurity (40), and intraventricular hemorrhage (41).

### Growth Measurements

Growth was compared between CG and IG using growth velocity and z score measurements. Weight (WT), length (L), and head circumference (HC) were recorded at birth and then weekly until discharge and plotted on the Olsen 2010 growth curves at each completed post-natal gestational week (42). Growth velocity for weight was calculated using the formula: [1000 × (Wn-W1)]/[(Dn-D1) × (Wn+W1)/2], where W is the weight in grams, D is day of life, 1 indicates the beginning of the time interval, and n is the end of the time interval in days (43). Growth velocity for length was calculated using the formula: Ln-L1/WKn-WK1, where L is the length in cm, WK is the week, 1 indicates the beginning of the time interval, and n is the end of the time interval in weeks. Growth velocity for the head circumference was calculated using the formula: HCn-HC1/WKn-WK1, where HC is the head circumference in cm, WK is the week, 1 indicates the beginning of the time interval, and n is the end of the time interval in weeks. WT, L, and HC Z scores were calculated at birth, 32- and 35-weeks' gestation (27, 42). Z scores were calculated using the Olsen 2010 growth curves (42).

### Skin Fold Thickness

Noninvasive measurement of body fat was done using skin fold thickness (SFT) obtained at discharge from the Level III NICU (44–47). Bicep, triceps, suprailiac, and subscapular SFT was measured in triplicate on the left side of the body using a standard skinfold caliper (Accu-Measure ® FitKid ^TM^ Caliper, Greenwood Village, CO). The skin was lifted with the thumb and index finger and the caliper remained in place until a constant reading was obtained and the mean of the triplicate measurement was used for analysis. The bicep SFT was measured 1 cm proximal to the crease of the elbow with the left arm extended. The triceps SFT was measured with the left arm flexed midway between the acromion and olecranon process. Subscapular SFT was measured just below the inferior angle of the left scapula at a diagonal in the natural skin fold. Suprailiac SFT was measured just superior to the iliac crest, along the left midaxillary line. Total SFT was the summation of all measured areas.

### Neurodevelopmental Follow-Up

Neurodevelopmental testing by Bayley III is routinely done in the Tufts Medical Center NICU Neurodevelopmental Follow-Up Clinic. We obtained 24 months +/- 6 months CGA Bayley III results for infants in our study that returned to the Follow-Up clinic for evaluation.

### Statistical Methods

Based on previously published data from our group on infant growth velocity at Tufts Medical Center (31) and on consideration of growth trajectories known to improve neurodevelopmental outcome (34), we performed initial power calculations to identify a 3.5 g/kg/day difference in growth velocity between CG and IG with 80% likelihood. The power calculation indicated that 48 subjects were needed to identify this difference with a *p* <0.05. We enrolled more than 48 patients to protect against drop out. The weight, length, HC growth velocities, z scores, skin fold thickness, and laboratory values (BUN, CO_2_, and creatinine) were summarized as mean and standard deviation and compared between groups using two-sided independent *t*-tests, Welch corrected. Milk protein content, total protein intake, and P/E ratio were evaluated by non-parametric ANOVA. The chi square test was used to assess for differences in categorical variables of maternal/infant demographics, mode of delivery, incidence of chorioamnionitis, preeclampsia, prenatal steroid administration, gestational diabetes, NEC, RDS, BPD, culture proven sepsis, ROP, IVH, and postnatal steroid use. Finally, we used a multivariate adjusted model to evaluate potential confounding factors including time to full enteral feeds, maximum caloric concentration of EBM, and formula intake and other covariates that met the *p* <0.1 threshold in univariate analyses. All statistical calculations were done using Prism, version 8.1 (Graph Pad Software, CA).

## Results

### Enrollment and Subject Demographics

The study population and study schematic are shown in Figure 1. A total of 82 eligible mother-infant pairs were identified between 2012 and March 2014. Seven infants died before enrollment, 4 were excluded for congenital anomalies, and 20 mothers refused consent. Of the 51 that were enrolled, one mother-infant pair in the treatment group withdrew 5 days into the study, leaving 20 control and 30 interventional group participants for completion of study.

Table 1 summarizes the characteristics of the study population. There were no statistically significant differences in characteristics of study population including weight, head circumference, and length at birth, except for differences in black racial distribution. IG had a higher number of whites (47 vs. 35%) and a higher number of Hispanics (37 vs. 25%), but no blacks. This racial distribution was statistically significant with *p* = 0.04. The comorbidities in both groups are also shown in Table 1. There were no significant differences between groups in the incidence of NEC, culture proven sepsis, BPD, ROP, and IVH. No postnatal steroids were received in either CG or IG. There were no differences in serum creatinine, BUN, or CO_2_ between the groups (data not shown).

**Table 1:** Subject characteristics.

**Characteristics**	**CG**	**IG**	**P (RR, 95 % CI)**
	**(*N* = 20)**	**(*N* = 30)**	
**Maternal**			
**Maternal age at delivery**			
<20 years - N (%)	3 (15)	2 (6.7)	0.63 (0.91; 0.74–1.12)
20–35 years - N (%)	12 (60)	21 (70)	0.67 (1.33; 0.62–2.87)
>35 years - N (%)	5 (25)	7 (23.3)	0.89 (0.98; 0.71–1.35)
**Race or ethnic group**			
Black - N (%)	4 (20)	0	0.04 (0.80; 0.64–1)
Hispanic - N (%)	5 (25)	11 (37)	0.58 (1.18; 0.82–1.72)
White - N (%)	7 (35)	14 (47)	0.60 (1.22; 0.77–1.94)
Other - N (%)	4 (20)	5 (16.7)	0.76 (0.96; 0.73–1.26)
Mode of delivery C-section - N (%)	14 (70)	22 (73.3)	0.80 (1.13; 0.46–2.75)
Steroid complete - N (%)	16 (80)	18 (60)	0.24 (0.5; 0.19–1.33)
Chorioamnionitis - N (%)	1 (5)	1 (3.4)	0.77 (0.99; 0.87–1.11)
Preeclampsia - N (%)	6 (30)	3 (10)	0.15 (0.78; 0.57–1.06)
Gestational diabetes - N (%)	1 (5)	3 (10)	0.91 (1.06; 0.9–1.23)
**Infants**			
Mean birth weight - g (± SD)	970.95 (± 217.9)	967.99 (± 245.4)	0.96
Mean birth head - cm Circumference (± SD)	24.8 (± 1.6)	25.1 (± 1.8)	0.49
Mean birth length - cm (± SD)	35.9 (± 2.4)	35.2 (± 2.7)	0.32
Mean GA in weeks (± SD; 95%CI)	27.3 (± 1.7; 26.5–28.1)	27 (± 1.7; 26.4–27.6)	0.54
Male sex - *N* (%)	10 (50)	17(56.7)	0.86 (1.10; 0.63–2.1)
NEC- *N* (%)	2 (10)	0	0.30 (0.90; 0.78– 1.04)
Culture proven sepsis- N (%)	2 (10)	1 (3.3)	0.72 (0.93; 0.79–1.09)
BPD- *N* (%)	3 (15)	6 (20)	0.94 (1.06; 0.82–1.37)
ROP- *N* (%)	6 (30)	11 (36.7)	0.85 (1.10; 0.74–1.64)
**IVH-** ***N*** **(%)**	6 (30)	6 (20)	0.64 (0.88; 0.62–1.23)
Grade 1 and 2	5	5	
Grade 3 and above	1	1	

### EBM Protein Content

**Figure 2:**
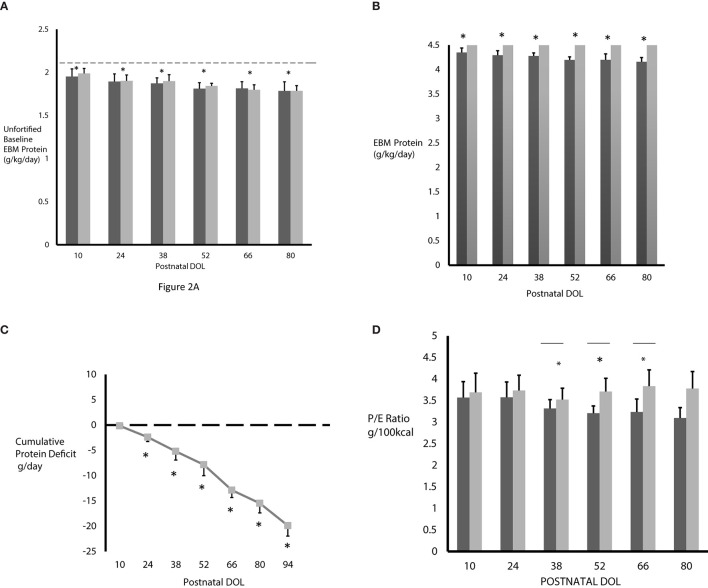
**(A)** The measured baseline EBM protein intake was significantly less in both CG (*n* = 3–20, dark gray bars) and IG (*n* = 5–30, light gray bars) compared to predicted protein content based on published mean EBM values of 2.1 g/kg/day from 150 mL/kg/day intake (horizontal dashed line). [*p < 0.0001 by ANOVA, predicted (dashed line) vs. measured baseline protein intake]. **(B)** The fortified EBM protein intake never reached the projected 4.5 g/kg/day in the control group on standard fortification (*n* = 3–20, dark gray bars) and progressively decreased over time. Protein intake in the interventional group on individualized fortification (*n* = 5–30, light gray bars) reached the goal of 4.5 g/kg/day. *p < 0.001 by ANOVA. **(C)** Without individualized fortification, subjects in CG accumulated a significant protein deficit over time. Protein deficit in IG is zero (dashed horizontal line). *p < 0.0001 by ANOVA at DOL 52, 66, 80; *N* = 3–20. **(D)** The P/E ratio was lower in CG (*n* = 3–20, dark gray bars) and progressively decreased over time. As expected, the P/E ratio was higher in IG (*n* = 5–30, light gray bars). *p < 0.05, unpaired *t*-tests, Welch corrected. All values are mean ± standard deviation and based on EBM intake of 150 mL/kg/day.

The measured unfortified baseline EBM protein content in CG and IG are shown in Figure 2A. Using the measured EBM protein content from the milk analysis, we calculated the actual unfortified baseline EBM protein intake in all subjects (Figure 2A, dark gray bar CG; light gray bar IG). The horizontal dotted line in Figure 2A represents the predicted protein intake based on published mean EBM protein levels. We found the measured baseline EBM protein content before fortification was similar between groups but significantly lower than published values in both CG and IG at DOL 10 (1.95 ± 0.09 g/150ml/kg/day and 1.99 ± 0.59 g/150mL/kg/day) and progressively decreased at each time point thereafter. This progressive decrease was significant (*p* < 0.0001) by one-way analysis of variance (ANOVA).

### Actual Protein Intake and Protein Deficit

The actual protein intake in CG and IG, with EBM protein fortification based on 150 mL/kg/day intake, is shown in Figure 2B. Compared to the IG, the protein intake in the CG was significantly lower at 4.35 ± 0.09 g/kg/day at DOL 10, decreased significantly at each time point thereafter, up to DOL 80, where it was 4.16 ± 0.09 g/kg/day, and never achieved the projected 4.5 g/kg/day protein intake. However, the protein intake in IG was much higher, as expected, and, due to using the actual level of protein in the EBM, reached the target of 4.5 g/kg/day at each time point. Using the measured and actual protein intake values shown in Figures 2A,B, we calculated the cumulative protein deficit in CG who received only standard fortification (Figure 2C). Use of standard fortification led to CG encountering a progressive and significantly different protein deficit at each postnatal time point studied compared to baseline values at DOL 10 (*p* < 0.0001) resulting in a cumulative protein deficit of 19.9 g by DOL 94 (*p* < 0.0001). As the IG subjects received individualized fortification, they did not actually accrue any protein deficit, as demonstrated by the dashed line in Figure 2C.

### Protein Energy (P/E) Ratio

Figure 2D shows the P/E ratio in CG and IG after fortification. At each time point, as expected, the P/E ratio was lower in CG on standard fortification. The difference in P/E ratio between the CG and IG was statistically significant at DOL 38, 52, and 66 (*p* < 0.0.05) and approached statistical significance on DOL 80 (*p* = 0.05).

### Growth Velocity

Growth velocity (Table 2) was calculated beginning at DOL 14 to eliminate variables associated with early postnatal fluid shifts and to address growth beginning at the time point that most infants would have regained birth weight. There was no difference in weight, length, or head circumference between CG and IG at DOL 14 (*p* value of >0.6 for wt, L and HC in CG vs. IG). This time point also coincides with milk analysis that began at DOL 10 and the time for when protein supplementation by milk analysis in IG was begun. When evaluating the whole cohort, we did not see significant differences over the first 6 weeks post-delivery in the weight, length, and head circumference growth velocities between groups. However, infants in IG born at <27 weeks gestation had a weight growth velocity over week 2–6 of life of 17.7 ± 3.5 g/kg/day and a head circumference growth velocity of 0.98 ± 0.22 cm/week whereas CG weight growth velocity was 15.6 ± 2.1 g/kg/day and head circumference growth velocity was 0.63 ± 0.6 cm/week. These values did not reach statistical significance, likely related to the small sample size in this gestational sub-group.

**Table 2:** Growth velocity.

**Growth measurement**	**CG Mean ± SD; 95%CI, (N)**	**IG Mean ± SD; 95%CI, (N)**	***P***
**Change in weight (g/kg/day)**			
24–29 6/7 weeks	17.1 ± 2.5; 16–18.2, (20)	16.8 ± 3.2; 15.6–18, (30)	0.7
<27 weeks	15.6 ± 2.1, 13.4–17.8 (6)	17.7 ± 3.5, 15.7–19.7 (14)	0.1
**Change in length (cm/week)**			
24–29 6/7 weeks	0.99 ± 0.47; 0.78–1.2, (20)	1.03 ± 0.4; 0.88–1.2, (30)	0.7
<27 weeks	0.98 ± 0.3, 0.66–1.3, (6)	1.1 ± 0.5, 0.8–1.4 (14)	0.58
**Change in HC (cm/week)**			
24–29 6/7 weeks	0.99 ± 0.8–1.2, (20)	0.87 ± 0.5; 0.65–1.1, (30)	0.4
<27 weeks	0.63 ± 0.6, 0.66–1.3, (6)	0.98 ± 0.2, 0.85–1.1, (14)	0.25

*HC, head circumference*.

### Z Score Data

**Figure 3:**
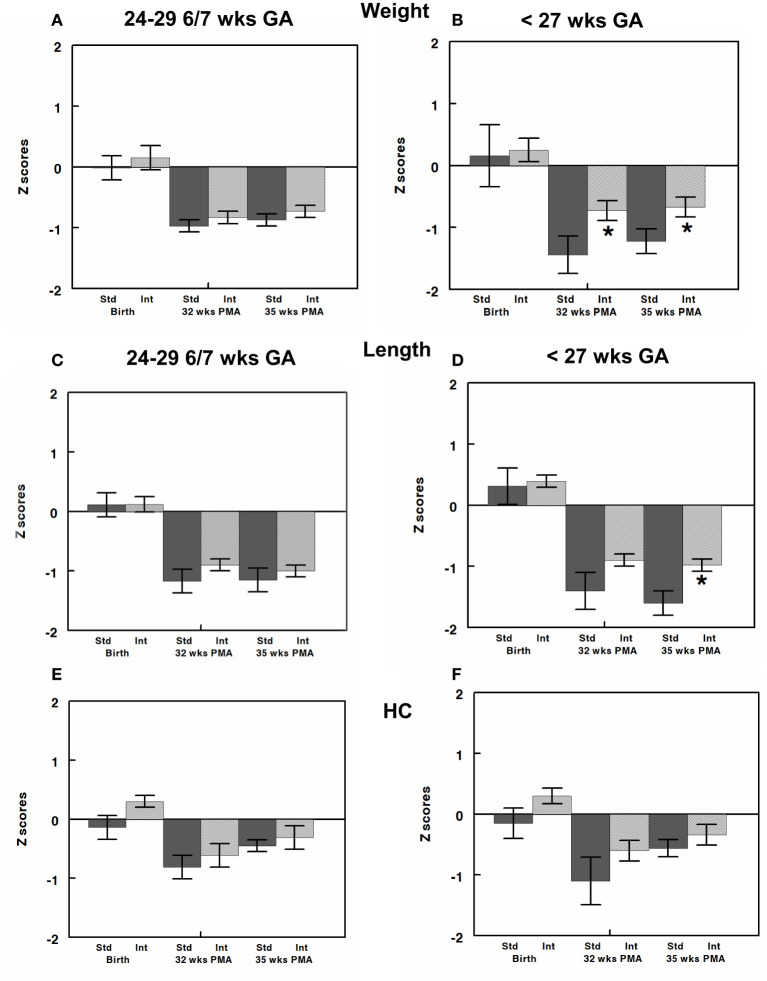
Z-scores for weight, length, and head circumference (HC) were calculated at birth and at 32- and 35-weeks post-menstrual age (PMA) and are shown for infants born at 24–29 6/7 **(A,C,E)** and those born at <27 weeks gestation **(B,D,F)**. All infants had similar z scores close to zero (50% on growth curve) at birth. Infants in both CG and IG exhibited some element of postnatal growth failure. However, z scores were greater in IG than CG and reached significance for weight z score at both 32 and 35 weeks and length z score at 35 weeks. All values are mean ± SEM; *N*, 6–30; *p < 0.05.

Weight, length, and head circumference z scores were determined at birth and at 32- and 35-weeks postmenstrual ages (21, 42, 48). Infants in CG and IG had similar weight, length, and head circumference z scores and, thus, growth percentiles, at birth (Figure 3). Despite our unit's nutritional protocol at the time of the study of beginning EBM fortification when enteral intake reaches 100 mL/kg/day both the CG and IG exhibited postnatal growth failure as shown by the negative z scores in Figure 3. While we did see postnatal growth failure in IG, the degree of postnatal growth failure was significantly less severe in IG for infants born at <27 weeks gestation when examined at 32- and 35-weeks postmenstrual age (Figures 3B,D,F). Specifically, for these infants, weight z score was significantly greater (Figure 3B) in IG compared to CG at 32 weeks (−0.73 ± 0.16 vs. −1.44 ± 0.3, *P* value 0.04) and 35 weeks (−0.67 ± 0.16 vs. 1.22 ± 0.2, *P* value 0.04). Length z score was significantly greater in the IG at 35 weeks postmenstrual age (−1.01 ± 0.1 vs. −1.66 ± 0.2, *P* = 0.02, Figure 3D). It is important to note the difference in growth percentiles for these z scores. For example, a z score of −1.6 corresponds to the 22%, z score of - 1.2 corresponds to the 28%, whereas a z score of – 1.0 corresponds to the 33% and that of – 0.6–0.7 corresponds to the 40–45% for any given age.

### Skin Fold Thickness

Skin fold thickness was used as a measure of lean body mass with lower skin fold thickness indicating leaner growth (Table 3). There was no significant difference between the mean gestational age of testing in CG (35 2/7 weeks ± 2 weeks 5 days) and IG (36 2/7 weeks ± 3 weeks 3 days). All areas measured for skinfold thickness were similar between CG and IG when evaluating the whole cohort. For infants born at <27 weeks gestational age, all skin fold thicknesses were lower in the IG, but this did not reach statistical significance for individually measured skinfold areas. However, total skin fold thickness (TSFT) (total of all areas measured) (47) was significantly reduced in infants in the IG born at <27 weeks gestation compared to CG (41.9 ± 2.2 IG vs. 46.8 ± 6.4 CG, *P* value 0.02).

**Table 3:** Skin fold thickness.

**SFT**	**CG**	**IG**	**P**
**Biceps (mm)**			
24–29 6/7 weeks	10.4 ± 1.1; 9.89–10.92, (20)	10.2 ± 0.6; 9.98–10.42, (30)	0.4
<27 weeks	11.5 ± 1.8; 8.6–14.4, (6)	10.2 ± 0.3, 9.8–10.5, (14)	0.3
**Triceps (mm)**			
24–29 6/7 weeks	10.9 ± 1.0; 10.43–11.37, (20)	10.5 ± 0.6; 10.28–10.72, (30)	0.08
<27 weeks	12 ± 1.4; 9.7–14.4 (6)	10.6 ± 0.56; 10.3–11.1, (14)	0.1
**Suprascapular (mm)**			
24–29 6/7 weeks	10.9 ± 0.9; 10.48–11.32, (20)	10.7 ± 0.7; 10.44–10.96, (30)	0.38
<27 weeks	11.6 ± 1.62; 9–14.1, (6)	10.6 ± 0.8; 10.1–11, (14)	0.3
**Suprailiac (mm)**			
24–29 6/7 weeks	10.8 ± 0.9; 10.38–11.22, (20)	10.5 ± 0.5; 10.31–10.69, (30)	0.14
<27 weeks	11.7 ± 1.66;9–14.3, (6)	10.5 ± 0.53; 10.2–10.8, (14)	0.25
**Total SFT (mm)**			
24–29 6/7 weeks	43.1 ± 3.7; 41.3–44.9, (20)	41.8 ± 1.9; 41.1–42.6, (30)	0.34
<27 weeks	46.8 ± 6.4; 36.6–56.9, (6)	41.9 ± 2.2; 40.7–43.1, (14)	0.02*

SFT, skin fold thickness; *Interventional compared to control group by unpaired T-test Welch corrected; all values are depicted as Mean ± SD; 95%CI, (N).

### Neurodevelopmental Testing

We observed a significant improvement in cognitive and motor Bayley III scores at 24 months CGA (Table 4A) in the IG compared to the CG in infants born between 24–29 6/7 weeks gestation. The trend in increased Bayley III scores remained for the infants at <27 weeks gestation but did not reach statistical significance likely related to the low numbers in this gestational subcategory that we were able to have return for follow up. There was no significant difference in sex, or the average gestational age at birth (27.2 ± 1.5 weeks CG vs. 26 ± 1.2 weeks IG), and birth weight (976 ± 158 g CG vs. 843 ± 173 g IG) of subjects that returned for developmental testing (Table 4B). While we had a lower percentage of infants return to follow up in the interventional group, this finding did not reach statistical significance. Additionally, we did not identify any statistically significant differences in comorbidities of infants in control and experimental groups that returned to neurodevelopmental follow up (data not shown).

**Table 4A:** Neurodevelopmental outcome at 2-years corrected gestational age in control and interventional groups.

**Bayley III scores, mean (SD) 2-years CGA**	**CG 24–29 6/7 weeks (*N* = 12)**	**IG 24–29 6/7 weeks (*N* = 10)**	**P value**	**CG <27 weeks (*N* = 3)**	**IG <27 weeks (*N* = 7)**	***P***
Cognition	88 (6.7)	101 (8.8)	0.004^*^	88 (12.6)	103 (8.6)	0.12
Language	84.5 (13)	91 (17.2)	0.45	85 (14)	97.3 (14)	0.40
Motor	89 (8)	95.9 (6.5)	0.046^*^	93 (9.6)	93 (5.4)	0.40

* *Interventional group compared to control group by unpaired T-test, Welch correction. All values are mean ± SD*.

**Table 4B:** Characteristics of infants who returned for neurodevelopmental follow-up.

	**Control**	**Interventional**	***P* Value**
2 year follow-up, N (%)	12 (60%)	10 (33%)	0.09
Corrected age (months), mean (SD)	23.7 (4.4)	22.9 (1.6)	0.68
Gestational age at birth, weeks, mean (SD)	27.2 (1.5)	26 (1.2)	0.06
Birth weight, grams (SD)	976 (158)	843 (173)	0.08
Male %	67	70	0.93

*No significant difference in prenatal steroid complete course, or comorbidities of sepsis, BPD, IVH, NEC, ROP by Fisher exact*.

### Potential Confounding Variables

We collected data on additional variable factors. We used a *p* value of <0.1 to determine potential confounding factors for a multifactorial analysis. This identified potential confounders, including day of life to reach full enteral feeds (150 mL/kg/day), number of days of formula use, maximum cal/oz (protein, fat, and carbohydrate calories per oz) of EBM received, and racial distribution. Formula use was defined as each day in which the subjects received >50% of formula for their total daily intake. One subject in IG received formula for more than 50% of their daily intake before DOL 42 compared to five in CG. Separate multivariate analyses were performed using each of the primary outcomes as the dependent variable and the combination of the study group and the above-mentioned potential confounding factors as the independent variables (Table 5). These analyses showed that none of these independent variables had a significant impact on the primary outcomes measured. The differences in racial distribution did not impact results based on multivariate regression analysis.

**Table 5:** Feeding patterns.

**Feeding pattern**	**Control group**	**Interventional group**	***P***
Day of life at full enteral feeds (Mean ± SD; 95% CI)	13 ± 3.2; 11.5–14.5 (*N* = 20)	15 ± 4.7; 13.3–16.8 (*N* = 30)	0.08
Days where formula use was >50% of feeding volume before DOL 42 (Mean ± SD; 95% CI)	8 ± 6.8; −0.5–16.5 (*N* = 5)	8 (*N* = 1)	0.09
Maximum cal/oz received (Mean ± SD; 95% CI)	26 ± 1.6; 25.3–26.8	27 ± 1.8; 26.3–27.7	0.05

## Discussion

Our study represents one of the few randomized trials on individualized protein fortification of expressed maternal breast milk in preterm infants while also analyzing the quality of infant growth and body composition and having information on neurodevelopmental outcome. To date the majority of studies on preterm infant expressed breast milk intake with macronutrient fortification have been observational or cohort studies (49). There has been an increased interest on the potential ease of analyzing human milk composition in clinical care of preterm newborns to more accurately provide a growing preterm infant adequate nutrition from expressed breast milk especially in relation to protein intake since protein composition of human milk decreases over time of lactation (3, 7, 13, 14, 17, 50–52). Technologies for human milk analysis are similar to those used in the dairy industry, where analysis of cow milk has been the routine for many years (52). The analyzer used in our study has acoustic spectroscopy and ultrasound technology that detects the differences in attenuation and transmission of the constituents within milk and uses this information to calculate protein content. This machine was the one used by US human milk banks at the time of our study.

Published data on EBM protein concentration of 1.4 g/dL has been used by manufacturers to develop the protein fortifiers used in neonatal intensive care units to nourish prematurely born infants (7). This concentration is based on EBM concentrations obtained at 2–3 postnatal weeks in a patient population where the mean gestational age was 33 weeks (range 27–37 weeks) (10–12). Underestimation of this baseline EBM protein may be partially responsible for the known poor postnatal growth and protein accretion in preterm infants (7, 21, 24) especially for infants in the gestational age range of those in our study. As we and others have previously showed, there is high variability in human milk protein composition between mothers, as well as with length of gestation, stage of lactation, and time of day, making standard calculations for protein intake less accurate (7, 8, 10–12, 14, 53–55). The decrease in breast milk protein content with length of time of lactation is nutritionally inadequate for the preterm infant in a metabolic window of high protein accretion and protein turnover.

Using individualized EBM analysis, we identified a significant difference between the predicted protein intake and the actual protein intake in infants born at <30 weeks gestation. Additionally, this difference significantly increased over time leading to a steadily increasing protein deficit and inadequate P/E ratio in CG (32, 56). For example, an infant born at 24–26 weeks gestation can have as much as a 19–20 g protein deficit by DOL 94 (as demonstrated by our data (Figure 2C). By fortifying the IGs EBM based on milk protein analysis, the target protein intake of 4.5 g/kg/day was reached at each time point, thus significantly increasing the P/E ratio in these infants and avoiding the protein deficit experienced by CG. While our study was not powered to detect a difference in lean body mass (decreased skin fold thickness), this increase in the P/E ratio may have improved lean body mass accretion and limited fat deposition in IG infants <27 weeks gestation at birth (25, 27). We chose in this study to focus on adjusting protein intake to optimize protein/energy ratio by using the milk analyses. While not also adjusting the fat and lactose concentrations based on the milk analyses in the interventional group may have led to some unknown variability in the intake of the other macronutrients and in the total energy intake, it is known that optimizing protein/energy ratio, the goal focused on in this study, is important to growth and development. While lactose and fat content change over length of time of lactation, fat and lactose concentration of EBM increases over the weeks of lactation but protein is the macronutrient that decreases progressively (10, 11). Satisfactory weight and length growth velocity (g/kg/day) and attainment of lean body mass improves neurodevelopmental outcome and decreases long-term morbidity and mortality (21, 27, 34, 57).

We prospectively sought to determine the difference in growth (g/kg/day), head circumference (cm/week), and length (cm/week) velocities between subjects receiving individualized EBM protein fortification based on milk protein analyses vs. subjects receiving standard EBM protein fortification (based on published information). We chose growth as our primary short-term outcome for the following reasons: (1) growth is an important factor impacting improved outcomes in ELBW infants; (2) it enabled us to use prior data from our NICU to power the study (31). We chose to stop calculating growth velocity at DOL 42 (6 weeks of age), based on this prior study and as the oldest infants in the cohort who were born at 29–30 weeks gestation at birth would have reached 35–36 weeks corrected gestational age at this time point and likely be ready for discharge (18, 22, 25, 26, 30–36). We were not able to show a significant difference in the growth velocity between the two study groups when evaluating the whole cohort (24–29 6/7 weeks) between 2 and 6 weeks of age. However, looking at the infants born at <27 weeks at birth, we saw a weight growth velocity of 17.7 g/kg/day in IG vs. 15.5 g in CG and head circumference growth velocity in IG of 0.98 cm/week vs. 0.63 in CG. Albeit this did not reach statistical significance due to the low numbers of subjects in this sub-cohort, it may have biologic importance. In evaluating our data, we discovered that the growth velocity in our preterm NICU population as well as for this gestational age subgroup had improved over time from our previously published data (31) that we used as the baseline growth velocity to design our study. This may reflect interim changes in the feeding practices in our NICU since the cohort in the Bartholomew study (31), with a focus on more aggressive use of parenteral nutrition in the first few days of life as well as EBM supplementation with human milk fortifiers and protein at lower enteral feeding volumes. Additionally, the subjects in our study were feed almost exclusively fortified EBM whereas another recent study looking at individualized fortification schemes had the majority of infants receiving formula supplementation (58). Predominant breast milk feedings in the early weeks after preterm birth is associated with improved neurodevelopmental outcomes in infants born at <30 weeks gestation (35). Finally, although our study was adequately powered for the overall study group, it had a small number of patients in the lower gestational age subgroup (<27 weeks gestation at birth). Future studies that focus on this youngest group of infants with adequate power may be more likely to identify significant improvement in postnatal growth when protein deficits are eliminated by individualized protein fortification.

In addition to evaluating growth velocity, we determined z scores at birth, 32- and 35 weeks postmenstrual age. Z score determination incorporated an analysis of each infant's growth based on growth curve data compared to a reference population and provided evaluation of growth at time points for some infants, especially the cohort born at <27 weeks gestation at birth, that were not captured by growth velocity determinations from 2 to 6 weeks of age. For example, an infant born at 24 weeks gestation would only be 30 weeks PMA at the time point of the 2–6 weeks of age growth velocity and, thus, an adequate view of extrauterine growth trajectory may not be completely evident at that time point. We learned several important points from this part of the analyses. First, despite improved growth and more progressive nutritional protocols in our NICU in recent years, both CG and IG exhibited postnatal growth failure although IG showed some significant improvements in z scores at 32 and 35-weeks postmenstrual age. Studies show that use of very early postnatal protein and calorie intake can positively influence growth in subsequent weeks, even when total nutrient intake is similar during these subsequent weeks (26, 34). This aspect may have been what allowed us to show a significant change in z scores for the youngest part of the cohort, whereas growth velocity determination at 6 weeks of age may not yet have detected these changes.

Studies also show that preterm infants with optimized head circumference growth have better neurodevelopental outcomes (59). The infants born at <27 weeks in the interventional cohort did have a trend toward increased head circumference growth velocity at 6 weeks of age and increased z score HC at 32 weeks and 35 weeks PMA, suggesting that optimized protein and P/E ratio may be associated with improved neurodevelopmental outcomes reflected by the higher Bayley III scores at 24 months. Improved postnatal linear growth has also been shown to predict neurodevelopmental outcome (27). Thus, it is interesting to note that the interventional group of infants born at <27 weeks had significantly greater length z scores at 35 weeks PMA.

There are limitations to this study. The study was powered for a 3.5 g/kg/day increase in weight gain velocity, which may have been overly optimistic. Consequently, the sample size is not adequate to detect smaller differences. This is especially true with regard to the <27-weeks GA subgroup. We used total protein for data analysis and did not convert it to bioavailable protein to account for the presence of non-protein nitrogen in human milk. While we did not compare the EBM protein values obtained from the milk analyzer to those obtained from standard reference laboratory methods, we did confirm reliable reproducible results with the milk analyzer prior to initiation of our study. The decision to evaluate protein content and other macronutrients along with the protein/energy ratio every 2 weeks was based on available information at the time of the study. However, changes in macronutrient content when milk was not measured was not known. Recent advances in milk analyzer technology, including low volumes of milk needed for analyses (~1 mL) may now allow for future studies to more frequently measure milk macronutrient content thus providing further improved nutritional care for preterm infants (60, 61). Devices that use air-displacement plethysmography technology to determine body composition (fat and fat-free mass) in infants appears to be a reliable instrument for assessment of body composition (62). Such a device was not available to us during our study and therefore we used traditional methods of SFT measurements to assess body composition. Despite these limitations, there are several important strengths to this study. These include the fact that it was a randomized, prospective, blinded study and that we used milk samples for analysis from a 24-h pool of EBM to measure protein content and also repeated this evaluation every 2 weeks during the study period. These measures prevented and controlled for nutrient variability that occurs over a 24-h period during lactation and over time of lactation (13). Additionally, the study was powered based on previously published growth data from our own institution. Further, our study population was composed of a homogeneous group of infants receiving mostly expressed breast milk as the base nutritional intake. Given that the standard nutritional approach in our NICU at the time of the study was potentially more progressive than used by others, the protein deficits we show may be further exaggerated in other nurseries that use slower advancement of milk supplementation schemes. This also lends support to the biologic and clinical importance of the information we present. We also looked at quality of growth by evaluating SFT and z scores in addition to growth velocity. Last, we were able to have some information on neurodevelopmental follow up within our study. While we had a 60% or less adherence to follow-up developmental testing, particularly in the interventional group (33% follow up), the infants who did return from the interventional group had improved neurodevelopmental scoring compared to the control group. Traditionally, families may be less likely to attend developmental follow up appointments if there are no concerns about development and learning. This may suggest that the infants lost to follow up, if able to be included, would have enhanced the improvements we saw in the interventional group.

In conclusion, we demonstrated that infants receiving standardized protein fortification strategies based on published breast milk protein levels develop significant protein deficits during a time in development when protein accretion is essential to organ development including brain development. Our data show that EBM protein fortification using EBM protein analysis is feasible and optimizes protein intake and P/E ratio eliminating this protein deficit. Additionally, we were able to show that later z-scores at 32–35 weeks gestation, especially for the most preterm infants, may be improved by such a protein fortification strategy and that these changes in practice may improve neurodevelopmental outcomes. Individualized EBM protein fortification is becoming an unavoidable tool in the nutritional management of preterm infants in the NICU environment. Larger studies are needed to confirm our results.

## Data Availability Statement

The raw data supporting the conclusions of this article will be made available by the authors, without undue reservation.

## Ethics Statement

The studies involving human participants were reviewed and approved by Tufts Health Sciences Institutional Review Board. Written informed consent to participate in this study was provided by the participants' legal guardian/next of kin.

## Author Contributions

MV and SK contributed to planning, execution of study, collection and evaluation of data, and writing of manuscript. AP contributed to execution of study and collection and evaluation of data for manuscript submission. EF and CS contributed to collection and evaluation of data and writing of manuscript. KH-W and HN contributed to planning of study, review of data, and writing of manuscript. All authors contributed to the article and approved the submitted version.

## Funding

This study was supported by Natalie V. Zucker Research Center for Women Scholars Grant.

## Conflict of Interest

The authors declare that the research was conducted in the absence of any commercial or financial relationships that could be construed as a potential conflict of interest.

## Publisher's Note

All claims expressed in this article are solely those of the authors and do not necessarily represent those of their affiliated organizations, or those of the publisher, the editors and the reviewers. Any product that may be evaluated in this article, or claim that may be made by its manufacturer, is not guaranteed or endorsed by the publisher.

## Abbreviations

CG: Control group
IG: Interventional group
EBM: Expressed breast milk
GA: Gestational age
PMA: Postmenstrual age.
